# Meet the Country Editor

**DOI:** 10.2174/1570159X2208240130154506

**Published:** 2024-01-30

**Authors:** Marco Scarselli

**Affiliations:** 1 Department of Translational Research and New Technologies in Medicine and Surgery,University of Pisa, Pisa, Italy

   Professor Marco Scarselli obtained his Ph.D in Neuropsychopharmacology from the University of Pisa (2003). He has worked as a Research Fellow at the Lab of Molecular Pharmacology, NIDDK, NIH (2003-2008) and as Junior Leader at the Lab of Nanoscale Biology of EPFL, Switzerland (2008-2011). Then, he moved back to the University of Pisa as Assistant Professor and from 2017 he is Associate Professor in Pharmacology. He has received national and international grants (*e.g*. Swiss National Science Foundation), and published many papers in the field of neuropharmacology in journals with high impact factor such as Nature Methods, PNAS, Molecular Pharmacology, Current Neuropharmacology, European Neuropsychopharmacology and Pharmacology & Therapeutics. He has received an Award (Guglielmo Scarlato) for his scientific contribution from Italian Parkinson Society.
